# Primary care provider experience and social support among homeless-experienced persons with tri-morbidity

**DOI:** 10.1186/1940-0640-10-S1-A64

**Published:** 2015-02-20

**Authors:** Erin J Stringfellow, Theresa W Kim, David E Pollio, Stefan G Kertesz

**Affiliations:** 1 Brown School of Social Work, Washington University in St. Louis, St. Louis, MO, 63130, USA; 2 Boston University School of Medicine, Boston, MA, 02118, USA; 3 University of Alabama at Birmingham, Department of Social Work, Birmingham, AL, 35294, USA; 4 School of Medicine, University of Alabama at Birmingham, Birmingham, AL, 35294, USA; 5 Clinical Addiction Research & Education Unit, Birmingham VA Medical Center, Birmingham, AL, 35233, USA

## Background

Persons living with mental illness, substance use disorder, and medical conditions, or “tri-morbidity,” have complex health needs. Tri-morbidity may be common among those who are homeless, and who face considerable obstacles to obtaining the high-quality, patient-centered health care and strong social support they need.

## Measures

Tri-morbidity was operationalized as meeting the following criteria: 1) probable mental illness or major psychiatric distress, based on reporting a diagnosis of post-traumatic stress disorder or schizophrenia, having ever taken psychiatric medication for a significant period of time, or a score of 30+ on the Colorado Symptom Index (range: 5–70) [1]; 2) lifetime moderate- or high-risk alcohol or illicit drug use, as measured using the Alcohol, Smoking, and Substance Involvement Screening Test (ASSIST) v. 3 [2]; and 3) reporting at least 1 of 14 physician-diagnosed chronic medical conditions.

Primary care experience was measured using the Primary Care Quality-Homeless (PCQ-H) tool (range: 1–4) [3]. Social support was measured using the "strong ties" scale (range: 3–15) [4], which queries the degree to which persons are bothered by not having a close companion, enough friendships, or people to whom they feel close.

## Methods

Patients (N = 601) from five geographically diverse primary care sites (four from the Department of Veterans Affairs [VA] and one health care for homeless program) were surveyed. Pearson’s chi-square, correlations, and *t*-tests assessed bivariate relationships. Multiple linear regression tested whether tri-morbidity predicted lower social support, compared to those without tri-morbidity, controlling for characteristics associated with strong ties.

## Results

Tri-morbidity was present in 39 percent of this sample of primary care-engaged, homeless, and formerly homeless persons (Table 1). Associated characteristics are shown in Table 2. Primary care experience was positive overall, as well as on all four subscales, and did not differ for persons with tri-morbidity (all p > .15). In the multiple regression model, persons with tri-morbidity had lower levels of social support (about 1.2 points on the strong ties scale; p < .0001) than those without tri-morbidity; controlling for financial hardship, minority, employment, and housing statuses; PCQ-H score; and having a live-in partner.

**Table 1:** Tri-morbidity among Primary Care-Engaged Formerly and Currently Homeless Persons (N = 601)

	N	%
Probable mental Illness or major psychiatric distress	428	71
Lifetime moderate- or high-risk alcohol or illicit drug use	357	59
At least 1 chronic medical condition	537	89
Tri-Morbidity (All of the above)	233	39

**Table 2:** Bivariate Comparisons of Characteristics by Tri-morbid Status (% and Means) (N = 601)

	Tri-morbid	Not Tri-Morbid
Primary Care Experience and Social Support		
Primary care provider experience (PCQ-H mean)	3.13	3.16
Social support (“strong ties” scale mean)	9.16	10.64*
Live-in partner (%)	10	10
Socioeconomic Status (%)		
Housed	54	68*
Working full- or part-time	13	22*
Hard to pay for basics	75	64*
Patient at VA Primary Care Site (%)	63	70
Demographics		
Gender (% male)	84	86
Minority (% non-white)	70	69
Average age	51.5	54.1*
*p < .05		

## Conclusions

Tri-morbidity was common in this sample of primary care-engaged formerly and currently homeless persons. Despite their increased complexity, the patient-reported primary care experience was not worse in the presence of tri-morbidity. Their lower social support, even compared to other homeless-experienced patients, might be relevant for primary care providers’ treatment plans.
